# First record of the monotypic genus *Acanopsilus* Kieffer, 1908 (Hymenoptera: Diaprioidea: Diapriidae) from the Eastern Palaearctic region

**DOI:** 10.3897/BDJ.4.e9572

**Published:** 2016-09-13

**Authors:** Chang-Jun Kim, Jong-Wook Lee

**Affiliations:** ‡ Korea National Arboretum, Pocheon, Korea, South; § Yeungnam University, Gyeongsan, Korea, South

**Keywords:** *
Acanopsilus
*, Belytinae, new records, new synonymy, Eastern Palaearctic

## Abstract

**Background:**

The monotypic genus *Acanopsilus* (Hymenoptera: Diapriidae), is hitherto known only from Europe, where it is widely distributed.

**New information:**

The genus is here recorded for the first time from South Korea and China, which constitutes the first formal record of the species from the entire Eastern Palaearctic region. A detailed redescription and photographs of *Acanopsilus heterocerus* (Haliday, 1857) are provided. Also, *Acanopsilus brevinervis* Kieffer, 1909 is proposed as junior synonym of *Anommatium ashmeadi* Mayr, 1856 (syn. nov.).

## Introduction

*Acanopsilus*, a monotypic genus of tribe Pantolytini (Diapriidae: Belytinae) was established (Kieffer 1908) based on the type species, *Acanopsilus clavatus* Kieffer. The genus is widely distributed in Europe, but has not yet been recorded outside the continent. Generally *Acanopsilus* is easily distinguished from other Pantolytini genera by the following characteristics: eyes bare; very long and slender scape with simple apical margin; femora slender; petiole cylindrical; antenna of female composed of 14 segments.

As a result of the present study, the known range of *A. heterocerus* is extended from Europe (Western Palaearctic) to South Korea and China (Eastern Palaearctic). A redescription of the species, along with figures of taxonomically important morphological features are provided.

## Materials and methods

The terminology used in the present study follows Nixon (1957) and Masner and García (2002). The images were taken with the aid of an Axiocam HRc camera mounted on a Discovery V20 stereomicroscope (Carl Zeiss, Oberkochen, Germany) and were produced with AxioVision40AC software (Carl Zeiss, Oberkochen, Germany). Final plates were prepared in Adobe Photoshop CS6 (Adobe Systems Incorporated, San Jose, United States of America).

The following abbreviations are used throughout the text: POL, distance between the inner edges of the two lateral ocelli; OOL, distance from the outer edge of a lateral ocellus to the compound eye; MT, Malaise Trap; YPT, Yellow Pan Trap.

## Taxon treatments

### Acanopsilus heterocerus

(Haliday, 1857)

Belyta heterocera Haliday 1857: 169.
Pantolyta heterocera : Ashmead 1893: 383.
Psilomma radiata Kieffer 1908: 424. Synonymized by Macek 1990.
Acanopsilus clavatus Kieffer 1908: 427. Synonymized by Macek 1990.
Psilomma radiata : Kieffer 1916: 415, 427.
Acanopsilus clavatus : Kieffer 1916: 428.
Pantolyta heterocera : Kieffer 1916: 433.
Acanopsilus clavatus : Ferrière 1930: 404.
Acanosema clavata : Nixon 1957: 21.
Acanosema heterocera : Nixon 1957: 23.
Acanopsilus clavatus : Kelner-Pillault 1959: 414.
Acanopsilus heterocerus : Hellén 1964: 57.
Acanopsilus clavatus : Kozlov 1978: 586.
Acanopsilus heterocerus : Kozlov 1978: 586.
Acanopsilus heterocerus : Macek 1990: 343, 345.
Psilomma radiata : Macek 1990: 343.
Acanopsilus clavatus : Macek 1990: 343.

#### Materials

- **Type status:** Other material. **Occurrence:** recordedBy: Jin-Kyung Choi; individualCount: 1; sex: female; lifeStage: adult; **Taxon:** scientificName: Acanopsilus heterocerus; **Location:** country: South Korea; stateProvince: Chungcheongbu-do; locality: Chungju-si, Suanbo-myeon, Samun-ri, Mt. Woraksan; verbatimCoordinates: 35°49'N, 128°04'E; **Identification:** identifiedBy: Chang-Jun Kim; dateIdentified: 2015; **Event:** samplingProtocol: Malaise trap; eventDate: 2013-07-17/08-12; **Record Level:** language: en; collectionCode: Hymenopteradiaprioidea; basisOfRecord: PreservedSpecimen
- **Type status:** Other material. **Occurrence:** recordedBy: Jin-Kyung Choi; individualCount: 1; sex: male; lifeStage: adult; **Taxon:** scientificName: Acanopsilus heterocerus; **Location:** country: South Korea; stateProvince: Chungcheongbuk-do; locality: Chungju-si, Suanbo-myeon, Samun-ri, Mt. Woraksan; verbatimCoordinates: 35°49'N, 128°04'E; **Identification:** identifiedBy: Chang-Jun Kim; dateIdentified: 2015; **Event:** samplingProtocol: Malaise trap; eventDate: 2013-06-16/07-17; **Record Level:** language: en; collectionCode: Hymenopteradiaprioidea; basisOfRecord: PreservedSpecimen
- **Type status:** Other material. **Occurrence:** recordedBy: Jin-Kyung Choi; individualCount: 1; sex: male; lifeStage: adult; **Taxon:** scientificName: Acanopsilus heterocerus; **Location:** country: South Korea; stateProvince: Gangwon-do; locality: Hongseong-gun, Mt. Maehwasan; **Identification:** identifiedBy: Chang-Jun Kim; dateIdentified: 2015; **Event:** samplingProtocol: Malaise trap; eventDate: 2015-06-27/08-01; **Record Level:** language: en; collectionCode: Hymenopteradiaprioidea; basisOfRecord: PreservedSpecimen
- **Type status:** Other material. **Occurrence:** recordedBy: Deuk-Soo Choi; individualCount: 4; sex: female; lifeStage: adult; **Taxon:** scientificName: Acanopsilus heterocerus; **Location:** country: China; stateProvince: Jirin; locality: Helong-si, Xicheng-jin, Mingyan-chon; verbatimCoordinates: 42°32'N, 129°00'E; **Identification:** identifiedBy: Chang-Jun Kim; dateIdentified: 2015; **Event:** samplingProtocol: Malaise trap; eventDate: 2009-07-03/10; **Record Level:** language: en; collectionCode: Hymenopteradiaprioidea; basisOfRecord: PreservedSpecimen
- **Type status:** Other material. **Occurrence:** recordedBy: Jong-Wook Lee; individualCount: 1; sex: male; lifeStage: adult; **Taxon:** scientificName: Acanopsilus heterocerus; **Location:** country: China; stateProvince: Jirin; locality: Helong-si, Xicheng-jin, Mingyan-chon; verbatimCoordinates: 42°32'N, 129°00'E; **Identification:** identifiedBy: Chang-Jun Kim; dateIdentified: 2015; **Event:** samplingProtocol: Malaise trap; eventDate: 2009-07-03/10; **Record Level:** language: en; collectionCode: Hymenopteradiaprioidea; basisOfRecord: PreservedSpecimen
- **Type status:** Other material. **Occurrence:** recordedBy: Jong-Wook Lee; individualCount: 1; sex: female; lifeStage: adult; **Taxon:** scientificName: Acanopsilus heterocerus; **Location:** country: China; stateProvince: Jirin; locality: Helong-si, Xicheng-jin, Mingyan-chon; verbatimCoordinates: 42°32'N, 129°00'E; **Identification:** identifiedBy: Chang-Jun Kim; dateIdentified: 2015; **Event:** samplingProtocol: Malaise trap; eventDate: 2009-07-10/27; **Record Level:** language: en; collectionCode: Hymenopteradiaprioidea; basisOfRecord: PreservedSpecimen
- **Type status:** Other material. **Occurrence:** recordedBy: Jong-Wook Lee; individualCount: 5; sex: female; lifeStage: adult; **Taxon:** scientificName: Acanopsilus heterocerus; **Location:** country: China; stateProvince: Jirin; locality: Helong-si, Xicheng-jin, Mingyan-chon; verbatimCoordinates: 42°32'N, 129°00'E; **Identification:** identifiedBy: Chang-Jun Kim; dateIdentified: 2015; **Event:** samplingProtocol: Malaise trap; eventDate: 2009-07-20/29; **Record Level:** language: en; collectionCode: Hymenopteradiaprioidea; basisOfRecord: PreservedSpecimen
- **Type status:** Other material. **Occurrence:** recordedBy: Jong-Wook Lee; individualCount: 1; sex: male; lifeStage: adult; **Taxon:** scientificName: Acanopsilus heterocerus; **Location:** country: China; stateProvince: Jirin; locality: Helong-si, Xicheng-jin, Mingyan-chon; verbatimCoordinates: 42°32'N, 129°00'E; **Identification:** identifiedBy: Chang-Jun Kim; dateIdentified: 2015; **Event:** samplingProtocol: Malaise trap; eventDate: 2009-07-20/29; **Record Level:** language: en; collectionCode: Hymenopteradiaprioidea; basisOfRecord: PreservedSpecimen
- **Type status:** Other material. **Occurrence:** recordedBy: Jong-Wook Lee; individualCount: 2; sex: female; lifeStage: adult; **Taxon:** scientificName: Acanopsilus heterocerus; **Location:** country: China; stateProvince: Jirin; locality: Helong-si, Xicheng-jin, Mingyan-chon; verbatimCoordinates: 42°32'N, 129°00'E; **Identification:** identifiedBy: Chang-Jun Kim; dateIdentified: 2015; **Event:** samplingProtocol: Malaise trap; eventDate: 2009-07-25/31; **Record Level:** language: en; collectionCode: Hymenopteradiaprioidea; basisOfRecord: PreservedSpecimen

#### Description

**Figure 1.**
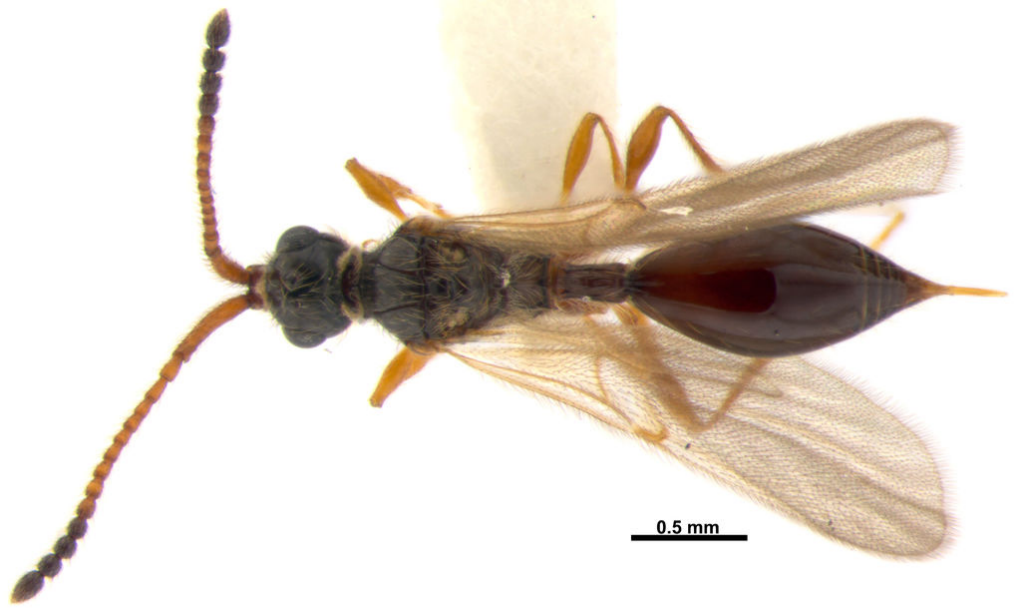
*Acanospilus heterocerus* Haliday, female. Habitus in dorsal view.

**Female** (Fig. 1​). *Head*. Head in dorsal view slightly wider than long (21: 20), slightly narrower than mesosoma (21: 23), OOL longer than POL (5: 3); occipital carina distinct, covered with dense whitish setae; vertex and frons smooth with sparse setae; tentorial pit large and deep; clypeus smooth and distinctly convex; mandibles crossed at apex, nearly symmetrical, left mandible with single inner tooth, right mandible with a pair of small teeth; head in lateral view slightly shorter than height (23: 21), with protrusive antennal shelf; eye large and bare, much shorter than height of head (1: 2), slightly longer than malar space (7: 6); antenna much shorter than body length (4: 7) and covered with short dense setae; antennal segments in following the proportions (length: width): 36:6; 9:5; 10:4; 6:4; 7:4; 6:4; 7:4; 7:5; 7:5; 7:6; 7:7; 7:8; 7:8; 11:8.

*Mesosoma*. Mesosoma much longer than width (15: 9); cervix with two large pits and bare in dorsal view; pronotal shoulders angled; epomia absent; mesoscutum convex and covered with long setae; notauli complete; humeral sulcus distinct; scutellum smooth, covered with long sparse setae and convex; anterior scutellar pit large and deep, transverse (5: 4), longer than remaining scutellar disc; posterior scutellar pits absent; mesosoma in lateral view clearly longer than high (25: 16); lateral part of pronotum smooth, bare and shiny; upper part of mesopleuron smooth, bare and shiny with deep sulcus under tegula, without sternaulus; lower part of mesopleuron smooth and covered with sparse setae; median keel of dorsellum prominent, tubercle-shaped; propodeum transverse; posterior margin of propodeum slightly emarginated; posterior transverse propodeal keel distinctly raised; median propodeal keel raised into ridge.

*Wing*. Fore wing with costal, subcostal, marginal and stigmal veins tubular; basal, cubital and medial veins pigmented; stigmal vein short, nearly perpendicular to the marginal vein, as long as post-marginal vein and half of marginal vein.

*Metasoma*. Petiole cylindrical in dorsal view smooth, bare, shiny, with irregular longitudinal keels, with long setae laterally and dense cushion of long setae ventrally; base of T2 with several costae, not angled in lateral view; following tergites with micropunctures medially and few long setae laterally; all sternites with sparse short setae.

*Color*. Head black; mesosoma and metasoma dark brown to blackish brown; antenna brown, except A11–A14 dark brown; legs, tegula yellowish brown; palps yellow.

*Measurements*. Head length 0.43 mm, width 0.47 mm; mesosoma length 0.80 mm, width 0.52 mm; metasoma length 1.45 mm; fore wing length 2.05 mm; total body length 2.68 mm.

**Figure 2.**
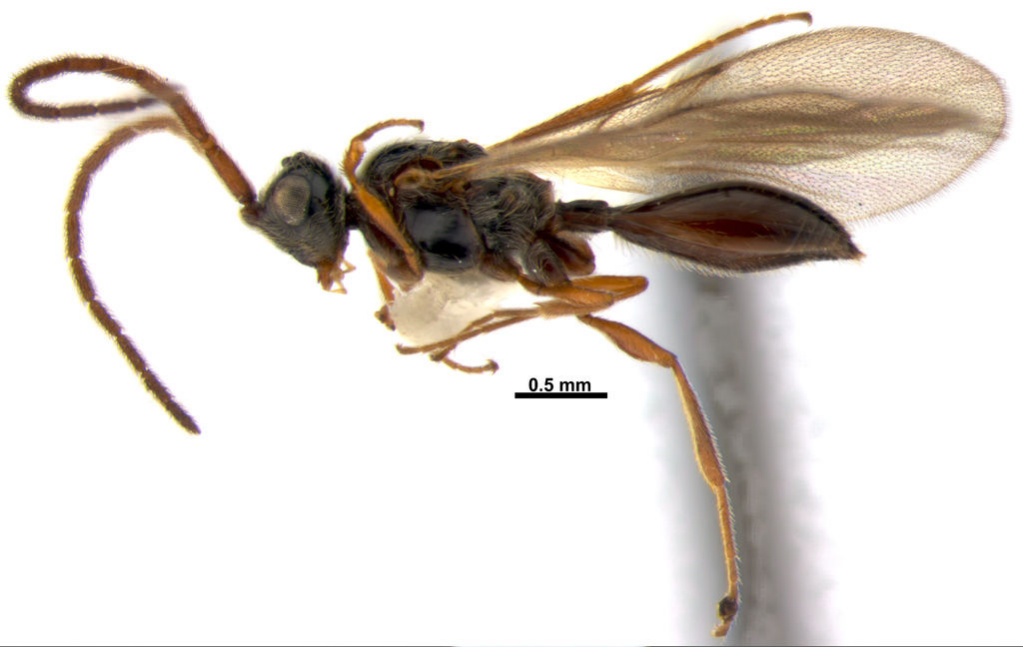
*Acanospilus heterocerus* Haliday, male. Habitus in lateral view.

**Figure 3.**
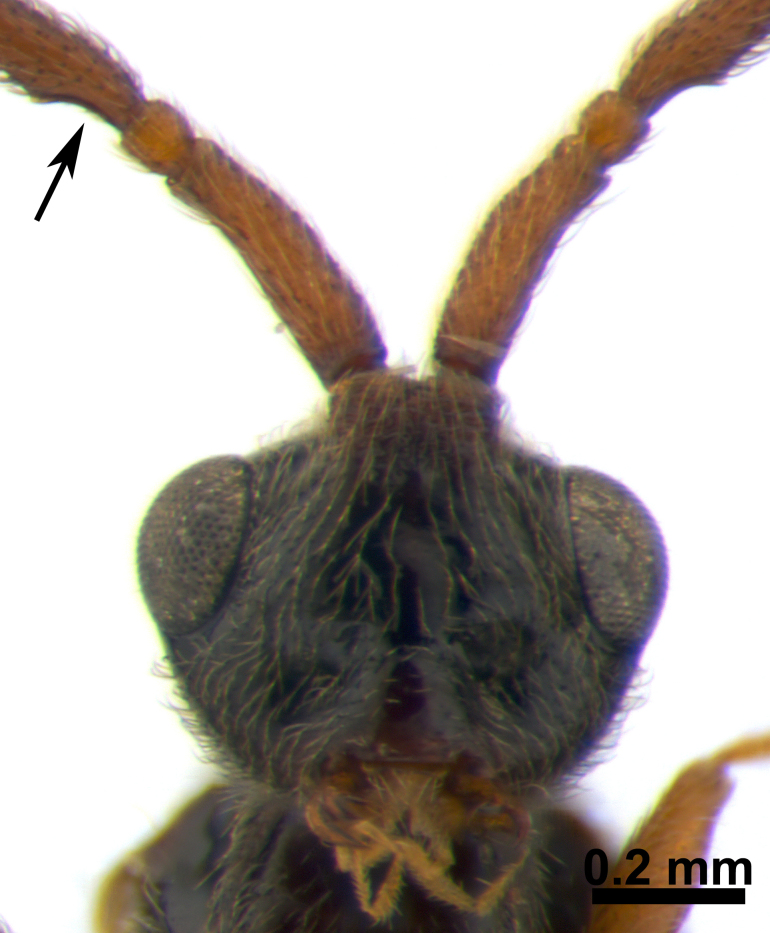
*Acanospilus heterocerus* Haliday, male. Head in frontal view.

**Male** (Fig. 2). Body length 3.33-3.60 mm. Similar to female, but antenna filiform, long and slender; A3 slightly emarginated basally (Fig. 3); antennal segments in following proportions: 30:8; 6:7; 27:6; 24:6; 24:5; 24:5; 24:5; 24:5; 21:5; 20:5; 19:4; 19:4; 18:4; 23:4.

#### Distribution

South Korea (new record), China (new record), widely distributed in Europe.

#### Host

Unknown.

#### Taxon discussion

The genus *Acanopsilus* was established by Kieffer (1908) based on the description of a single species. Subsequently he (Kieffer 1909) described a further three species: *A. arcuatus*, *A. laticeps* and *A. brevinervis*. The first two were synonymised with *Acanosema nervosum* (Thomson 1858) by Macek (1990). Macek (1990) also expressed his opinion that *A. brevinervis* was a synonym of *Anommatium ashmeadi* Mayr 1904 but he did not formalise the synonmy, therefore the genus *Acanopsilus* technically contains two species viz. *A. brevinervis* Kieffer, 1909 and *A. heterocerus* (Haliday, 1857) (Johnson 1992, Hymenoptera Online 2016). After we examined the type specimens of *Acanopsilus brevinervis* and *Anommatium ashmeadi*, we agreed with Macek’s (1990) opinion. *Acanopsilus brevinervis* is here in synonymized with *Anommatium ashmeadi*​. Hence, *Acanopsilus* is regarded as monotypic.

## Supplementary Material

XML Treatment for Acanopsilus heterocerus
